# A Deep Learning Approach for Table Tennis Forehand Stroke Evaluation System Using an IMU Sensor

**DOI:** 10.1155/2021/5584756

**Published:** 2021-04-09

**Authors:** Sahar S. Tabrizi, Saeid Pashazadeh, Vajiheh Javani

**Affiliations:** ^1^Department of Computer Engineering, Faculty of Electrical and Computer Engineering, University of Tabriz, Tabriz 5166616471, Iran; ^2^Department of Information Technology, Faculty of Electrical and Computer Engineering, University of Tabriz, Tabriz 5166616471, Iran; ^3^Department of Sport Management, Faculty of Physical Education & Sport Sciences, University of Tabriz, Tabriz 5166616471, Iran

## Abstract

Psychological and behavioral evidence suggests that home sports activity reduces negative moods and anxiety during lockdown days of COVID-19. Low-cost, nonintrusive, and privacy-preserving smart virtual-coach Table Tennis training assistance could help to stay active and healthy at home. In this paper, a study was performed to develop a Forehand stroke' performance evaluation system as the second principal component of the virtual-coach Table Tennis shadow-play training system. This study was conducted to show the effectiveness of the proposed LSTM model, compared with 2DCNN and RBF-SVR time-series analysis and machine learning methods, in evaluating the Table Tennis Forehand shadow-play sensory data provided by the authors. The data was generated, comprising 16 players' Forehand strokes racket's movement and orientation measurements; besides, the strokes' evaluation scores were assigned by the three coaches. The authors investigated the ML models' behaviors changed by the hyperparameters values. The experimental results of the weighted average of RMSE revealed that the modified LSTM models achieved 33.79% and 4.24% estimation error lower than 2DCNN and RBF-SVR, respectively. However, the ${\bar{R}}^{2}$ results show that all nonlinear regression models are fit enough on the observed data. The modified LSTM is the most powerful regression method among all the three Forehand types in the current study.

## 1. Introduction

COVID-19 (Corona Virus Disease 2019) has been spreading rapidly since December 2019. The World Health Organization (WHO) declared COVID-19 as a pandemic on 11 March 2020 [1]. COVID-19 is highly contagious; it has led to acute respiratory disorder and severe cases, and it has caused multiple-organ failure and, thus, significant mortality. This disease has caused panic and has become a worldwide nightmare. According to WHO reports, the first case of COVID-19 was reported in Wuhan, China [2]. This disease has caused a devastating effect on the well-being and health of the global population and hurt their mental health. The side effects of this virus influence the economics and safety of billions of people as well. These effects have surprised the world.

Quarantine and social distance are severe steps in the fight against COVID-19. However, one of the most significant points that may be forgotten during a crisis like the lockdown days of COVID-19 is mental health problems. In this situation, people's everyday activities are confined to a small area. These limitations may lead to increased restlessness, stress levels, and adverse consequences of physical inactivity. Behavioral Immune System theory indicates that people can develop negative cognitive and negative emotions to protect themselves in these situations [3, 4]. Besides, based on perceived risk [5] and stress [6] theories, public health emergencies cause more negative emotions. However, in a short time, these emotions keep people away from the disease [7]. On the other hand, when a crisis period is becoming unpredictable, these negative emotions may decrease people's immune function and devastate their balance of physiological mechanisms [3].

Being active “#BeActive” and healthy at home “#HealthyAtHome” are two campaigns presented by WHO to encourage people to fight together against the COVID-19 and stay healthy. WHO recommended 30 min of physical activity for all healthy adults and 60 min for kids in the crisis days [8]. Inactive situations caused by lockdown affected ordinary people's lives and seriously affected athletes' lives. Athletes who need to practice and keep their performance high during the pandemic of COVID-19 have to practice more than the WHO recommended duration. In most sports fields, doing practice needs coach supervision, particularly in individual sports that require hand equipment such as racket sports and throwing sports.

Shadow practice/exercise, or so-called shadow-play, is a sports training technique that involves repeating actions and imitating a sport's specific skill [9, 10]. This effective technique can help players, particularly novice players, learn the proper form of a sport's style and agility [9]. In Table Tennis, which is a popular sport that requires hand equipment, the shadow-play technique improves the players' strokes performing skills [9]. The practice of Table Tennis strokes without the ball is the definition of Table Tennis shadow-play. Not only does shadow-play develop players' stroke performing skills, but it can train the players' brains in terms of the racket's proper position and the feeling of how the correct stroke technique should be played [10, 11]. Shadow-play can be valuable if applied under the supervision of trained coaches [9].

Nevertheless, due to numerous iterations of shadow-play, this technique needs professional coaching resources. The coach must allocate a significant amount of time, which is not available on these quarantine days. An accessible, practical, and intelligent system with minimal cost could be an appropriate solution to assist the trainee automatically during these days.

Machine learning (ML) algorithms were inspired by human brain structure and can play the role of a human in most of the real-life problems (in limited conditions for specific reasons), such as the education sector [12], industrial sector [13], health sector [14], and transportation systems [15, 16]. On the other hand, sensors as cutting-edge technologies are responsible for reliable and accurate information gathering regarding peoples' activities. Thus, a combination of sensor technology and ML algorithms could be an appropriate solution to overcome the real coach's absence during these crisis days (in limited conditions for specific purposes).

### 1.1. Motivation

Developing a shadow-play assistance system of Table Tennis as a virtual-coach system, with three main components, namely, (1) shadow stroke classification, (2) shadow stroke qualification, and (3) player guidance, is the perspective of the authors [17]. Table Tennis Topspin, Push, and Basic Forehand strokes [18] are considered the motions whose quality scores are evaluated and estimated in the current study.

**Figure 1:**
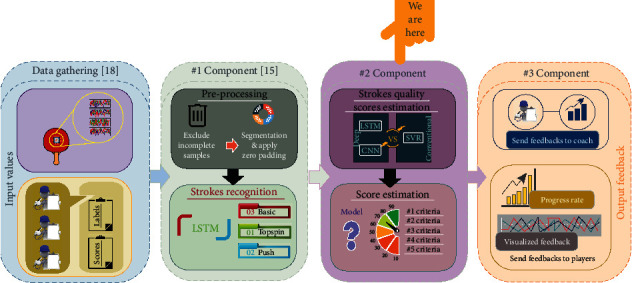
The architecture of the Table Tennis shadow-play virtual-coach system.

We completed the shadow stroke classification phase as the first component of the desired system [17]. Thus, we take the next step to create the virtual-coach Table Tennis shadow-play training system. The system is capable of providing a personalized training system for players, particularly novice ones. Generally, the individual sports training qualities that require equipment used by the hand depend on the equipment's movements and orientations' correctness. Thus, identifying the appropriate multivariate regression ML methods to apply to the second component of the system is considered in this study.  Figure 1 depicts the architecture of the virtual-coach system.

### 1.2. Contribution

The three main contributions of this paper are as follows: (1) the authors introduce the self-collected dataset, which is made publicly available at [19], consisting of segmented sensor data (accelerometer, gyroscope, magnetometer, and Euler angles) from one miniaturized, inexpensive, and nonintrusive inertial sensor (BNO055) mounted on a standard Table Tennis racket. Moreover, the study's data collection phase's details and steps were released in the authors' last work [20]. (2) We examine and compare the ML models' performance when their parameters were tuned based on the dataset. The results of parameter-tuning attempts are publicly available at [21]. (3) We determine a multivariate regression model that takes the sensory data as input and estimates the score of performed stroke qualities at the output.

### 1.3. Outline

The rest of the paper is organized as follows. Related research is summarized in Section 2.  Section 3 includes the methodologies of the study, and Section 4 covers the experimental setup. Experimental results are presented in Section 5. Conclusion and future studies are given in Section 6.

## 2. Related Studies

Machine learning (ML) algorithms make significant progress in solving, estimating, and predicting problems of Activity of Daily Life (ADL), sports activity analysis, and health and disease issues. In this section, the related works of estimation of ADL and sport activity are presented. The studies are considered based on the developed applications, types of sensing modalities, and applied algorithms.

The task of estimation in Activity of Daily Life (ADL) necessitates the use of sensors, which has been well addressed in previous studies and commercial products. Numerous studies have involved various sensor modalities in capturing human motion or biometric information. These studies have addressed the subject of human daily life activity estimation with appropriate performance.

Brophy et al. [22] presented a robust heart rate estimation study with a photoplethysmography as a body-worn sensor. The Convolutional Neural Network with Regression (CNNR) was applied to estimate the heart rate [22]. Barut et al. [23] introduced a new dataset consisting of human activity types and activities' intensities. They introduced a novel multitask Long Short-Term Memory (LSTM) network to recognize human activities and estimate their passions. A single ActiGraph GT9X as a wearable sensor measured human activities [23]. Zihajehzadeh and Park [24, 25] developed a walking speed estimation system with a wrist-worn inertial measurement unit (IMU) and Gaussian process regression. Ahad et al. [26] conducted three IMU sensors placed on the top of a backpack or waist-belt to estimate age and gender. The underlined multitask LSTM and temporal Convolutional Neural Network (CNN) revealed good results [26]. Zerkouk and Chikhaoui [27] proposed a method based on LSTM to estimate older people's abnormal behaviors. They used SIMADL [28] dataset in their experiments. A novel symptom-based “Unified Parkinson Disease Rating Scale” estimation system was presented by Hssayeni et al. [14]. They used two wearable sensors with a dual-channel LSTM [14].

Sports activity and performance estimation systems, particularly for individual sports, are another popular area considered in this section. Numerous studies have involved using cameras or various sensor modalities or a combination of both methods to capture and measure athletes' motions. These studies have addressed the subject of sports activities analysis and estimation with appropriate performance.

Dadashi et al. [29] developed a swimming velocity estimation system with a single IMU to help coaches provide impressive guidance to trainees. Martin et al. [30] presented a novel vision-based stroke classification system of Table Tennis with a new Twin Spatiotemporal CNN algorithm. Lim et al. [31] developed a coaching assistant system of Table Tennis with three body-worn IMUs. LSTM networks (unidirectional and bidirectional) with probabilistic features were applied to classify the strokes. They claimed that the developed systems' promising results based on high dimensional time-series data collected via the sensors could help the players practice Table Tennis [31]. The Gradient Boosted Regression Trees algorithm was applied to a new fatigue prediction model for runners based on a single wrist-worn IMU [32]. Support Vector Regression (SVR) was introduced as a more reliable athletes' performance prediction model [33]. Another severe problem in most sports fields, particularly football, is the recovery time after an undiagnosed injury. This problem was considered by [34]. He presented a model to predict “recovery time” with neural network based on the professional football club of Tottenham Hotspur members' data. Likewise, [34, 35] considered football players' injury prediction problems. Their developed system used k-nearest neighbors (KNN) and SVR in two phases. A similar study was presented by [36] based on an Australian football club players' GPS and accelerometer data with linear regression and SVM. Wiik et al. [37] developed a system based on the performance monitoring system's data for athletes (pmSys) dataset with LSTM to predict future peeks in a soccer player's readiness-to-train.

By considering the type of data, Postorino and Versaci [16] underlined the fact that, in the case of the uncertain or linguistic-based data, their proposed ellipsoid-shaped fuzzy model with the neuro approach (ellipsoid-shaped + ANFIS) outperforms the conventional fuzzy systems. Their research results show that the developed model is applicable in real-life applications with low computational complexity.

The applications developed by Zhang [38], Martin et al. [30], Lim et al. [31], Blank et al. [39], and Liu et al. [40] are the only related academically published papers in the area of Table Tennis. These studies, except Lim et al. [31], only considered Table Tennis stroke classifications and did not consider the Table Tennis training issue. Moreover, Zhang [38] and Martin et al. [30] did not use any sensors as the sensing modalities; thus, their developed systems could be considerably expensive to implement in practical conditions. Both Lim et al. [31] and Liu et al. [40] developed body-worn sensor systems, but both designs were considerably complex for the players to set up and wear. Blank et al. [39] used an object sensor as a sensing modality, which places a sensor on the Table Tennis racket. It seems that their proposed system is more practical to set up in real conditions for stroke classification. On the other hand, the system developed by Lim et al. [31] is the only academically published one in the area of Table Tennis training. They claimed that the developed system' promising results indicated that it would be beneficial for Table Tennis players to practice their strokes. However, until now they [31] have not published any quantitative results. In addition, the orientations and movements of the racket that are crucial in Table Tennis practice and training were neglected in Lim et al.'s study [31].

Literature review revealed that, in the case of conventional regression models, SVR is mostly applied. In the deep learning techniques, the LSTM and CNN are most commonly used to estimate time-series sensory data [41]. Besides, it has revealed that, in the literature, stroke quality evaluation of Table Tennis Forehand based on a single object sensor has yet to be considered. Because of this deficiency in the literature, the proposed study considers a single object sensor as the sensing modality in addition to a high-performance multivariate regression algorithm to estimate the scores of quality of Table Tennis Forehand strokes.

## 3. Materials and Methods

The structure of object sensors as the data collection tool and the basics of the regression algorithms (shallow and deep models) are briefly described in this section.

### 3.1. Object Sensors

As one of the most common and popular sensing modalities, object sensors are widely used to develop sensor-equipped devices to measure motions and orientations. For inferring human motions or activities, object sensors are mounted on a specific object(s) to measure its movements or orientations [42]. Accelerometers and gyroscopes are two conventional sensors that have traditionally been utilized in the robotics and human motion analysis fields. The low-cost and miniaturized features are crucial for accelerometers and gyroscopes for developing state-of-the-art systems such as navigation, gestural control, motion measurement and detection, personal health, and augmented reality. The Microelectromechanical System (MEMS) is the combination of electrical systems with the mechanical structure on a micrometer scale, which could be a proper solution for the issue mentioned above [43]. MEMS devices have provided low prices with high-quality sensing capabilities. The design of MEMS is a multidisciplinary research endeavor that uses the concepts and methods of physics, mathematics, and engineering. Various technologies for designing MEMS have been presented. Using the electrostatic field and investigating its influence on the geometric curvature of the membrane for MEMS devices and the numerical model of the curvature of the membrane and the electrical magnitude has been investigated [44, 45].

Di Barba et al. [44] have proposed a framework of one-dimensional membrane Microelectromechanical System (MEMS) theory. In the steady-state case, they have formalized the problem of existence and uniqueness of a solution for the membrane deformation of electrostatic actuation. They investigated the presence of the solution using Schauder–Tychonoff fixed point theorem. Using a numerical approach for the reconstruction of the membrane profile in one-dimensional MEMS devices has been accomplished. The applied voltage produces an electrostatic field whose direction is, locally, orthogonal to the membrane's surface [45]. Angiulli et al. [45] rewrote the condition of the solution's existence in the form of an equivalent dual problem. The dual problem has been solved using the numerical shooting approach, the method of secants, and the Matlab solver for ordinary differential equations. The authors have investigated the necessary condition for the convergence guarantee of the numerical method.

The IMU is developed by various technologies such as MEMS. Typical sensors used in the IMU include gyroscope and accelerometer, which can measure angular velocity and directional acceleration, respectively, and involve magnetometer and barometer sensors. The low weight and small size of IMUs are the advantages that MEMS offers. Based on these advantages, IMUs are used in many applications [46], particularly in motion measurement.

Moreover, IMUs can produce excellent accuracy in the short term and long term by utilizing filters and external references [46]. A literature review [47, 48] revealed that IMUs could be used to determine the attitude of an object(s) and track its positions. The Attitude and Heading Reference System (AHRS) is a sensor fusion algorithm built based on the IMU to enable the combination of multiple-sensor measurements to yield one orientation measurement. All released signals from the sensors (gyroscope, accelerometer, and magnetometer) are fed to an AHRS algorithm to combine them and produce a measure of orientation relative to magnetic north and gravity. Furthermore, Euler angles (roll, pitch, and yaw angles) are computed based on the IMU sensors' measured signals. Euler angles illustrate rotations around three orthogonal axes (*x*, *y*, and *z*). Another specification of the IMUs is the Degree of Freedom (DoF). DoF is a typical designation for IMU sensors, which signifies the released number of the sensors' independent signals. Many 9-DoF sensors are available in most modern commercial IMUs, consisting of a 3-axis gyroscope, a 3-axis accelerometer, and a 3-axis magnetometer [49]. AHRS algorithms are mostly based on Fuzzy Logic, Kalman filter (KF), and Artificial Neural Network (ANN). KF has been used in commercial IMUs [38].

### 3.2. Regression

The object sensors provide a large amount of sensory data during the measurement of motions or orientations. Conventional ML algorithms such as SVR were considered in most previous studies that addressed sensory data-based estimation systems with high accuracy. Deep learning is a new paradigm of machine learning. The multiple process layers of deep algorithms provide facilities to extract and analyze big-data information without manual feature engineering [50]. CNN and Recurrent Neural Network (RNN) are the most well-known deep models used for sensor-based approach activity and motion estimation. Unlike other conventional models, we can apply SVR to big data without feature selection or reduction processes, like deep ones. SVR and deep models can learn high-level features by training, and they are more feasible to perform in real-life problems [51, 52]. Both model types' performance has been assessed in terms of the accuracy of the applied algorithms under actual test conditions [53].

#### 3.2.1. Long Short-Term Memory (LSTM)

A RNN has been proposed to address recognition and estimation problems in time-series datasets [54, 55]. Besides, as one of the typical deep methods, RNN utilizes the temporal correlations between neurons to process natural language and recognize speech [56, 57]. When the standard RNN input data is a long sequence, the model is faced with the exploding problem and gradient vanishing challenge due to the training phase. The LSTM method was introduced by Hochreiter and Schmidhuber [58] to overcome these challenges. LSTM cells are combined with the standard RNN method by replacing its hidden layer and playing the memory unit's role through gradient descent [57, 59, 60]. After that, it is trained using the backpropagation algorithm.

#### 3.2.2. Convolutional Neural Network (CNN)

CNN has a multilayer feed-forward neural network architecture. CNN was inspired by the biological visual system [61]. A convolutional layer(s), pooling layer(s), fully connected layer(s), and output layer comprise the structure of the CNN; they are stacked together and can be trained entirely as a whole. Consequently, using a backpropagation algorithm and appropriate optimized training parameters with a proper optimizing algorithm is a common way of achieving this. In the one-dimensional convolutional layer, to compute the convolution between $\overset{⟶}{x}$ and $\overset{⟶}{f}$, $\overset{⟶}{f}$ as a convolutional filter slides along $\overset{⟶}{x}$. By computing dot product in each step and calculating the convolutional layer [62], we get

(1) $$\begin{array}{c} \overset{⟶}{x\;}\in R^{n},\\ \overset{⟶}{f\;}\in R^{m},\\ c\in R^{n-m+1},\;\text{where }c_{i}=f^{T}x_{\left[i:i+m-1\right]}. \end{array}$$

Hyperbolic tangent, Sigmoid, and Rectified Linear Unit (ReLU) are three common activation functions typically applied to the convolutional output values. ReLU is a simple thresholding operation defined as ReLU(*x*)=max(0 · *x*).

The main goal of the pooling layer is to summarize and reduce the obtained representation. The maximum or average of the data's small rectangular blocks is applied in more than one convolutional choice. The fully connected layer's primary goal, which follows the pooling layer(s), is to compute this vector as its output layer. All additional features are stacked into this vector [61].

#### 3.2.3. Support Vector Regression (SVR)

One of the popular supervised learning algorithms is the Support Vector Machine (SVM) [52]. Vapink [63] introduced SVM as a high-performance and flexible method that has been successfully applied to various regression problems and classifications. Support Vector Regression (SVR) uses the basic idea of SVM. It is a useful tool that applies as a real value estimate function [64]. The kernel function *k*, regularization parameter *γ*, and penalty parameter *c* are the configurable SVR parameters that have to be set in advance to achieve the highest performance [65, 66]. Linear, Radial Basis Function (RBF), Sigmoid, and Polynomial are the SVR possible kernel functions [64]. Unlike SVM, SVR computational complexity is independent of the input data space's dimension. This capability is one of the main advantages of SVR. High prediction accuracy and generalization capability are two other main advantages of SVR.

## 4. Experimental Setup

A set of experiments was applied to the self-collected dataset to evaluate the proposed method's performance. As seen in Table 1, the current study consists of four main sections, namely, (1) hardware setup, (2) data gathering [20], (3) preprocessing [17], and (4) processing. The first three phases are in charge of sample collection and preparation. The processing phase of the study is responsible for identifying an appropriate regression method. The proposed regression method will be applied to estimate the value of the performed strokes' scores quality based on stroke evaluation metrics of Table Tennis. Generally, regression methods, particularly deep ones, are sensitive to the parameter settings. The Random Selection method is applied to sample selection. Besides, the parameter-tuning phase of the methods is also accompanied by verification. Table 1 reports the framework of the experiments in the four main steps in detail.

### 4.1. Hardware Setup

As seen in Figure 1, the self-collected dataset contains two parts: (1) automatically captured data and (2) manually recorded data. The automatically captured data contains the IMU sensors' time-series signals for each stroke measured by the developed racket. The IMU includes a gyroscope and accelerometer, measuring angular velocity and directional acceleration, respectively, and a magnetometer. Furthermore, Euler angles (roll, pitch, and yaw angles) are computed based on the IMU sensors' measured signals. Euler angles illustrate rotations around three orthogonal axes (*x*, *y*, and *z*). The captured time-series data were transmitted via a USB cable to a laptop computer running the data gathering software. The received measured data at any time *t* contains four groups of data as follows:

(2) $$\begin{array}{c} {\text{data}}_{t}=\left\{\text{Acc}\cdot \text{Gyro}\cdot \text{Mag}\cdot \text{Euler}\right\},\\ \text{Acc}=\left\{{\text{ACC}}_{x}\cdot {\text{ACC}}_{y}\cdot {\text{ACC}}_{z}\right\},\\ \text{Gyro}=\left\{{\text{Gyro}}_{x}\cdot {\text{Gyro}}_{y}\cdot {\text{Gyro}}_{z}\right\},\\ \text{Mag}=\left\{{\text{Mag}}_{x}\cdot {\text{Mag}}_{y}\cdot {\text{Mag}}_{z}\right\},\\ \text{Euler}=\left\{{\text{Roll}}_{x}\cdot {\text{Pitch}}_{y}\cdot {\text{Yaw}}_{z}\right\}. \end{array}$$

In the following subsection, the reasons behind the selection of the appropriate sensor, the selected sensor placement, and the sensor calibration are described.

#### 4.1.1. Selecting an Appropriate Sensor

Vision-based sensing modalities are widely used to develop sports motion analysis systems [67]. Lighting, natural and indoor environment challenges, vision blocking, expensive sensing tools, and complex setup are some of the challenges faced by vision-based systems. Miniaturized, inexpensive, lightweight, and easy mountable specifications are characteristics of object sensor modalities, particularly IMU series sensors. IMUs could be used as an alternative for the vision-based sensing modalities used in most motion detection and orientation measurement cases with high performance. In a similar study [43], a 9-DoF BNO055 factory-calibrated IMU sensor with KF was used to measure Table Tennis Forehand strokes time-series data without noise. A basic description and the key features of the BNO055 and its integrated sensors are given below. Linear acceleration, Euler angles, gravity, rotation vector, heading, and quaternion matrix comprise the BNO055 fused sensor output data. It can hold three advanced sensors in one device: a triaxial 16-bit gyroscope, a versatile, a full performance geomagnetic sensor, and a triaxial 14-bit accelerometer with a 100 Hz sampling rate. Detailed specifications of the module are available in [68]. In this study, the sampling rate of BNO055 is set to 70 Hz.

#### 4.1.2. The Sensor Placement

**Figure 2:**
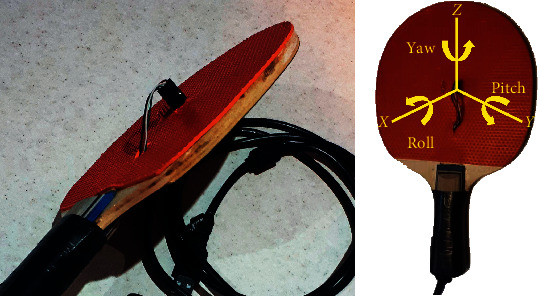
(a) The developed racket and (b) the determined IMU sensor placement [20].

Not only the appropriate type of sensor is essential, but proper sensor placement is also crucial. A literature review revealed that individual sports' motion and orientation data requiring hand equipment were captured from the sensors mounted on the equipment [69]. Usually, the object sensor does not directly interact with users. Thus, to collect raw data from the sensors, two critical issues have to be considered: (1) the developed objects would have to be user-friendly; (2) the sensors placed on the item would have to be nonintrusive [57]. The BNO055 was mounted on various standard Table Tennis racket positions to determine the sensor's best position. Based on the nature of the Forehand movements, the blade's center was empirically determined as the appropriate sensor placement. The rubber secures the IMU.  Figure 2 shows the developed Table Tennis racket and the sensor placed on it.

#### 4.1.3. The Sensor Calibration

Although the IMU is a factory-calibrated sensor, the authors had observed the BNO055 calibration when the samples were collected. The IMU's released calibration values range from 0 to 3 for each embedded sensor separately. The “not calibrated” status is shown by zero value, and the “fully calibrated” status is indicated by value three. The sensor's datasheet describes the calibration guidance steps when the sensor calibration status shows zero value [68].

### 4.2. Data Acquisition

Due to the lack of available Table Tennis strokes' sensory data with their quality scores dataset, the authors collected the data. The data acquisition protocols were defined before obtaining the data. Subsequently, based on the defined protocols, samples were gathered, scored, and labeled. All details of the data gathering phase are published in the authors' recent article [20], and the collected samples are made publicly available in [19]. In the following subsections, these steps and characteristics of the participants are briefly described.

#### 4.2.1. Participants' Characteristics

Three groups of participants were involved in this study, namely, (1) novice players, (2) professional players, and (3) coaches. The I.R. Iran Table Tennis Federation (TTF) Ethics Committee approved the current study, and all participants delivered their written agreement in advance. The novice players' group consists of eight mixed-gender first-year students of the Physical Education and Sport Science Department at the University of Tabriz, with height of 161 ± 4 cm and age of 19 ± 4 years, who volunteered as novice players in this study. The professional players' group consisted of eight mixed-gender experienced and highly national and international ranked players, with height of 171 ± 8 cm and age of 28 ± 4 years. The coach group participating in this study consists of three high national and international ranked Table Tennis coaches who were recommended and introduced by the I.R. Iran TTF in East Azerbaijan.

#### 4.2.2. Defining Data Acquisition Protocols

The players used the IMU mounted racket when samples were collected. Three main protocols have been determined for the players' groups in advance. Protocol 1: all players were familiar with the preparatory condition for Forehands training (the positioning, the accurate way to grip the racket, and posture of the Forehand strokes). Protocol 2: the players waited five seconds between each repetition. Protocol 3: the players were familiar with the Forehand shadow-play stroke evaluating and scoring metrics. Each subject's performance was evaluated, scored, and labeled by the coaches placed around Table Tennis's table. The coaches are placed around the table to observe each player's performance and score the performed stroke qualities separately. The places were determined based on the coaches' best observation conditions and the elimination of blind spots associated with the players' arms. Capturing order of the samples is as follows: (1) the players started performing strokes, (2) they were paused for five seconds by the coaches at the end of each stroke performing, (3) the sensory data was stored by the software into a single CSV file, and (4) the players' performance was labeled and scored by the coaches manually. A group of 16 players comprised of both genders participated in the creation of the self-collected dataset samples. All professional players performed each Forehand stroke type 45 times. Overall, 1080 samples were captured for the professional group. In the case of the novice group, 648 samples were collected from 8 novice players.  Table 2 explains the collected data statistical specifications briefly. As seen in Table 2, the self-collected dataset is not balanced.

#### 4.2.3. Labeling and Scoring Samples

As seen in Figure 1, in each sample collecting session, besides the automatic captured sensory data of performed strokes, the labeling and scoring processes were applied. These processes generated three separate lists of the strokes' labels (B, T, or P), along with the scores (0–100 percentage) of the stroke evaluation criterion (*C*_1_, *C*_2_, *C*_3_, *C*_4_, *C*_5_) manually assigned by the coaches. Converging and diverging angle of the racket gripping during the performance (*C*_1_), forward swing (*C*_2_), follow-through (*C*_3_), appropriate speed of the racket movement (*C*_4_), and performed stroke general quality (*C*_5_) were applied as the evaluating and scoring criteria for Table Tennis Forehand training in this study [38, 70]. The coaches contributed to the supervision of the data collection phase. They supervised each player's stroke performing speed and preparatory conditions of the Forehand strokes. Regarding the performance scoring method, in some sports like gymnastics and ice skating, for each criterion, the average of the coaches' set scores was determined as the final score for all performed strokes of the players.  Table 3 depicts the schema of the proposed method to create the dataset.

### 4.3. Data Preprocessing

Due to the mounted sensor's short-time usage during the data collection, the sensor drift rate and sensor aging effect are negligible [17]. Nevertheless, we must consider the sensor drift rate and aging factors for the sensor's long-term usage. In the current study, the preprocessing phase was applied to the measured sensory data.

This phase includes three main steps: (1) eliminating incomplete samples, (2) segmenting time-series signals, and (3) normalizing the segmented signals. The preprocessing phase results have already been published in the authors' previous paper [17].  Table 4 shows the basic information of the dataset after the preprocessing step. Consequently, the dataset consists of 1525 samples of performed strokes. Each collected sample consists of three different types of data: (1) the sensory data of the racket movements and orientations with 840 features (840 features: 12 (value of data_*t*_) × 70 (length of segment)); (2) the final value of the players' strokes quality scores based on the criteria released from the scoring section; and (3) the label of the performed strokes (B, T, or P).

### 4.4. Processing

We applied the setup and parameter settings of both network groups (shallow and deep) after the preprocessing phase. The models' performance metrics are also explained in this section. As seen in Figure 1, the strokes that have been detected in the classification phase [17] are ready for evaluation. An accurate assessment and scoring of the sport performance are essential to give guidance and feedback for the players, particularly novice ones [71, 72]. Moreover, this feedback will help prevent a false stroke pattern from institutionalizing the player's performances. Therefore, to modify this faulty pattern, a lot of time and costs are needed. In this regard, identifying an appropriate regression ML algorithm to estimate the strokes' quality scores was considered in this section. The ML algorithms were trained and tested based on the coach evaluation scores to assess the quality of the performed strokes. These scores were collected and calculated during the data acquisition phase [20]. Thus, to evaluate each type of Forehand strokes (Basic, Topspin, and Push), three separate ML models are needed. To benchmark the study, a conventional regression method (SVR) and two commonly used deep neural networks (LSTM and CNN) were utilized [41]. We took a sequence of the features *x*_1_,…, *x*_840_ as input data for the ML algorithms representing the sensory-data dataset. The models' outputs estimate the strokes' quality scores (*C*_1_, *C*_2_, *C*_3_, *C*_4_, *C*_5_).

#### 4.4.1. Network Setup

To avoid overfitting through the training phase, the models were benchmarked and their performances were compared fairly; for both groups (shallow and deep), *K*-fold cross-validation (*K* = 4) was applied to evaluate the testing and training performance of the modified methods. The proposed application has been implemented for various low power platforms.

All models are trained offline on a computer equipped with 3.7 GHz i7-8700k core processors, 32 G RAM, and NVIDIA 1080 Ti GPU. The trained models are capable of exporting to other platforms to deploy applications, particularly mobile applications. To implement the LSTM and 2-Dimensional Convolutional Neural Network (2DCNN) estimators, we applied the Python library *Keras* with the backend of *TensorFlow*.

#### 4.4.2. Parameter Setting

The proposed deep and shallow methods contain a set of parameters that have to be set in advance before being trained. We can say that fine-tuning models may achieve better results. On the other hand, there is no universally accepted method for selecting the appropriate parameters' values. The chosen methods' parameters are tuned carefully by applying the validation dataset and a trial and error strategy.

(i) For the SVR, the Radial Basis Function (RBF) was selected as its kernel function. We validated the soft-margin parameters with parameter *c* and parameter Gamma (*γ*) in the range (10^0^ to 10^−7^). For all three types of strokes, the optimum RBF-SVR *c* and *γ* parameters were chosen as 10^0^ and 10^−7^, respectively.
(ii) In the case of the deep models, we validated the parameters with the following values:
  1. The Epoch number in range (100, 250, and 500).
  2. The batch size in range (10, 50, 100, 500, and 1000).
  3. The number of the layers (LSTM and convolutional layer) in range (1, 2, and 3).
  4. The number of filters in range (2^4^ to 2^8^).
  5. The rate of dropout in range (0.1, 0.2, 0.3,…, 0.9).
  6. The number of the dense layers (fully connected layers) in range (1, 2, and 3).
  7. The number of neurons in the dense layer in the range (2^3^ to 2^6^).

**Figure 3:**
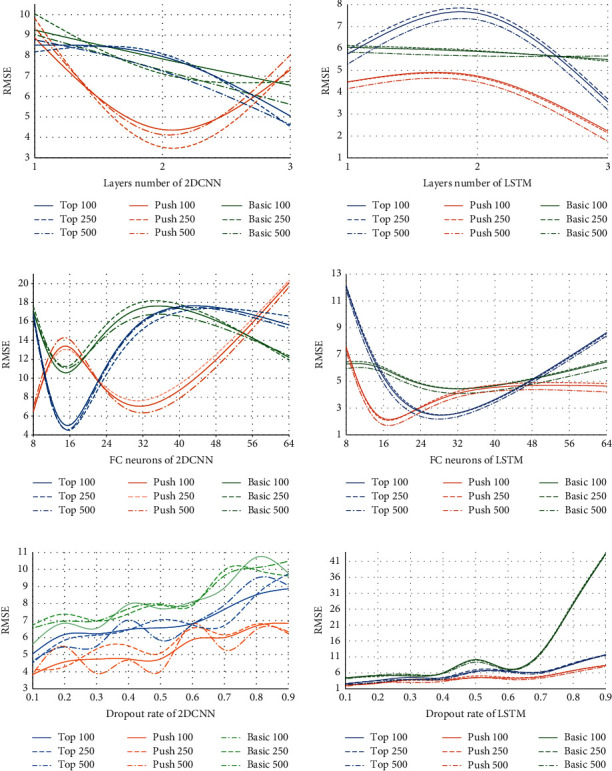
The deep models' behavior according to three different hyperparameters: (a) number of the hidden layers; (b) number of the neurons in the FC layers; (c) dropout rate.

The number of LSTM's layers, or so-called memory cells, affects the LSTM models' ability to memorize, erase, and ignore new inputs. In the CNN case, the convolutional layer is responsible for the feature extraction from the input data. Like LSTM, CNN's performance depends on the number of the convolutional layers and the neurons' number in the dense (fully connected) layers. To avoid overfitting the dropout rate, it represents a technique that ignores a specific rate of arcs.  Figure 3 depicts the change of the loss function values, Root Mean Square (RMSE), for a different set of the three hyperparameters. The figure shows the test phase of the LSTMs and 2DCNNs for 100, 250, and 500 Epochs as an example.  Figure 3(a) depicts the models' different behaviors on the testing dataset with different numbers of the memory cells and convolutional layers of 1, 2, and 3. The best performance of the LSTM models is reached with 3 memory cells. In the case of the 2DCNN, the best performance was gained with three layers for Basic and Topspin and two convolutional layers for the Push model.  Figure 3(b) demonstrates the effect of the number of neurons in the fully connected (FC) layer from 2^3^ to 2^6^. In this figure, the Basic and Topspin models of the LSTM with 32 neurons and the Push LSTM model with 16 neurons reached the highest performance. In 2DCNN, the Basic and Topspin models with 16 neurons got the best performance, and for the Push, the number of neurons is 8.  Figure 3(c) indicates the LSTM and 2DCNN models' configuration with different dropout rate values from 0.1 to 0.9. It can be observed that the best performances of both LSTM and 2DCNN models are released when the rate is 0.1.

(i) The best performance of the LSTMs on three separate datasets is reached in the following cases:
  - Basic Forehand: The proposed LSTM model involves three LSTM layers with 512, 256, and 128 filters, respectively, and 100 Epochs with batch size of 50. The dropout rate is 0.1. It consists of two fully connected layers with the size of 32 neurons.
  - Topspin Forehand: The proposed LSTM model comprises three LSTM layers with 1024, 512, and 256 filters, respectively, and 100 Epochs with batch size of 100. The dropout rate is 0.1. It consists of two fully connected layers with 32 neurons.
  - Push Forehand: The proposed LSTM model consists of three LSTM layers with 1024, 512, and 256 filters, respectively; 100 Epochs with 50 batch size; dropout rate of 0.1; and two fully connected layers with the size of 16 neurons.
(ii) Like LSTM, the 2DCNN parameters are validated with the seven parameters within the same range. In the pooling layer case, max pooling operation was applied for all three types of Forehand models. The best performance of the 2DCNNs on three separate datasets are reached in the following cases:
  - *Basic Forehand*: The proposed 2DCNN model consists of three convolutional layers with 4, 8, and 16 filters, respectively; 500 Epochs with batch size of 10; a 0.1 dropout rate; and two fully connected layers with the size of 16 neurons.
  - *Topspin Forehand:* The proposed 2DCNN model involves three convolutional layers with 8, 16, and 32 filters, respectively; 250 Epochs with batch size of 10; a 0.1 dropout rate; and one fully connected layer with the size of 16 neurons.
  - *Push Forehand:* The proposed 2DCNN model comprises two convolutional layers with 8 and 16 filters, respectively; 250 Epochs with batch size of 10; a 0.1 dropout rate; and three fully connected layers with the size of 8 neurons.

We determined the batch size, the number of Epochs, and the number of hidden layers after several empirical tuning attempts for better regression performances [73]. ReLU was chosen as the activation function for both proposed deep models. Moreover, for deep models, the “RMSprop” was selected as an optimizer. In our study, the results of feature selection attempts revealed that these techniques removed critical and crucial information for stroke evaluation. For all proposed models, regardless of the model type (deep or shallow), we chose RMSE as a loss function. The online available index reports all possible computations results for the deep models [21]. The results of parameter-tuning attempts for the LSTM and 2DCNN on the three types of Forehand dataset released 48600 possible models.

#### 4.4.3. Compute Performance Metrics

Choosing the appropriate ML performance metrics is crucial for the ML pipeline. We addressed RMSE as an evaluation metric to evaluate the performance of Multiple Input Multiple Output (MIMO) estimation problems [74]. We set it as the loss function in all three models. The benchmark methods performances are compared primarily based on the RMSE. Two other conventional metrics addressing the estimation problems, Mean Absolute Error (MAE) and Mean Absolute Percentage Error (MAPE), are analyzed. Moreover, based on the nature of the dataset, the Adjusted Coefficient of Determination (${\bar{R}}^{2}$) is considered to compare the goodness-of-fit of the developed regression models for the dataset.

### 4.5. Models' Performance Evaluation

This section explains the RMSE, MAE, MAPE, and ${\bar{R}}^{2}\;$for quality evaluation of the Topspin, Push, and Basic strokes of both groups of methods (shallow and deep) using the Table Tennis Forehand stroke self-collected dataset.

#### 4.5.1. Comparing the Models' Performances

Based on the nature of the Forehand strokes' racket movements, we empirically determined the center of the surface as the appropriate sensor placement location in this study. Thus, the collected data was acquired by mounting the sensor in the center of the racket's surface.

The same experiments have to be applied to the same dataset [75] to evaluate various models' performance.  Table 5 shows the optimum performance for each of the nine models to estimate the performed strokes' scores quality based on the stroke evaluation metrics. As outlined in Table 5, we observed that ${\bar{R}}^{2}$ values of all proposed models are over 90.0% on the dataset. The revealed results showed that all nonlinear regression models are fit enough on the observed data. It leads the authors to examine the models' performance results in the next step and continue the comparison.

In the models' performance, the proposed three LSTM models with three different parameters' values achieved the lowest estimation error rate compared to the other six types of models.  Table 6 provides statistical information on the regression outputs generated by RBF-SVR, LSTM, and 2DCNN models. The mean and standard deviation (STD) values of the LSTM models' output data were much smaller than the other models. Note, it was found that the LSTM models with three different configurations generated the output result with the smallest deviation (Basic-STD = 3.78, Top-STD = 4.12, and Push-STD = 2.76) for each stroke, compared to 2DCNN (Basic-STD = 6.82, Top-STD = 6.96, and Push-STD = 3.42) and RBF-SVR (Basic-STD = 4.19, Top-STD = 4.73, and Push-STD = 3.13).

## 5. Results

The performances of the three nonlinear conventional regression models, LSTM, 2DCNN, and RBF-SVR, for Table Tennis Forehand strokes' quality estimation problem were compared based on the self-collected dataset. These regression models' performances were analyzed by using RMSE, MAE, MAPE, and ${\bar{R}}^{2}$. For fair comparison, 4-fold cross-validation technique was applied to all models. All model parameters and hyperparameters were tuned in advance to find the appropriate configuration of the models. As outlined in Table 5, all three proposed regression models' training and testing performances revealed that overfitting did not occur. Regarding the weighted average of RMSE, MAPE, and MAE on the testing dataset, the proposed LSTM model had the lowest error value, and the SVR-RBF model had the second lowest error rate (see Table 7). The proposed LSTM models' weighted average of RMSE is 3.39, MAPE value is 0.05, and MAE value is 2.86, which are lower than RBF-SVR and 2DCNN models' results. On the other hand, the RBF-SVR performance is so close to the optimum LSTM. According to study [17], based on the number of RBF-SVR parameters, the model parameters tuning is significantly easy to choose. Thus, the model is far less challenging to develop. Besides, the SVR model theoretically gives better performance in small-scale training datasets [65]. However, the LSTM provides better performance in large-scale ones, as in this study's collected data. This means that the proposed LSTM models outperform both RBF-SVR and 2DCNN models.

The experimental results of the different parameter-tuning attempts indicated that the proposed LSTM could be a practical and feasible method. Moreover, the weighted average of ${\bar{R}}^{2}$ value, 99.7% of all three proposed LSTM models with a different configuration, revealed that the LSTMs are more fit than the other models on the observed data.

Besides, the LSTM model can consistently and considerably outperform the other two models of the study. In other words, the modified LSTM is the most powerful regression method among all three Forehand types in the current study.

## 6. Conclusions and Future Work

As time continues to pass, day by day, the new life paradigms that the pandemic COVID-19 has caused are clarified dramatically. Quarantine and social distance have significant effects on people's daily lives and personal and social relations. This disease has caused a devastating impact on their well-being and mental health. The literature review revealed various studies related to COVID-19 disease treatment, recognition, diagnoses, estimation, etc., based on the ML techniques. However, one of the most significant points that may be forgotten during the crisis lockdown days of COVID-19 is mental health problems. Psychological and behavioral evidence suggests that home sports activity could reduce negative moods and anxiety during these days. Thus, a low-cost, nonintrusive, and privacy-preserving (not visual) smart sports training assistance solution could help to stay active and healthy at home. The answer could be highly affordable for the general population. In the big picture, the authors' main aim is to develop the virtual-coach Table Tennis shadow-play training system. The system is capable of providing personalized training for players, particularly novice ones. In the current study, the proposed LSTM, as the appropriate method, was applied to the third step of the system to estimate the performed Table Tennis strokes' scores qualities based on the study's five evaluation metrics. This study's primary purpose was to develop an evaluation system of Table Tennis Forehand stroke for the three types of Forehands (Basic, Topspin, and Push) by considering both practical and technical aspects. By considering the practical aspect, object sensor sensing modality was used in this study to develop the data collection tool. As a miniaturized and inexpensive sensing tool, the IMU was embedded in the center of the standard Table Tennis racket's surface to measure movements and orientations of strokes automatically. Unlike other sensing modalities and approaches, inertial-sensor-equipped devices provide measurements by considering privacy (not visual) and nonintrusive issues. According to the selected sensing modality and based on the selected IMU's specifications, the developed racket makes the solution appropriate for cost-effective and nonintrusive assisted Table Tennis shadow-play training.

Moreover, as another practical aspect of the study, the coaching group who participated in this study set scores of the performed strokes' quality and labeled them manually. Considering the technical aspect, it is also crucial that the proposed Forehand quality estimation models achieve high estimation performance. Three different nonlinear regression methods were applied and compared based on two deep models, namely, LSTM and 2DCNN, and one shallow model, RBF-SVR. The experimental results revealed that the three LSTM models with different configurations constitute the most powerful regression method. The weighted average of all three LSTM models' performance of RMSE is 3.39, MAPE is 0.05, MAE is 2.86, and ${\bar{R}}^{2}$ is 99.7%. Results show that the LSTM model performs very well in evaluating Forehand strokes, including Topspin, Push, and Basic Forehands. The study's technical and practical aspects demonstrate that the proposed model has a high potential to be successfully applied in the second principal component of the Table Tennis shadow-play systems (see Figure 1). Unlike other similar studies that have used professional cameras [30, 38] or multiple wearable sensors [31, 40], the developed system only uses one object sensor to measure the Forehands' signals. The configuration and installation of vision-based sensing modality would cause the shadow-play assistance solution to be expensive, making it less affordable for the general population. The selected object sensor sensing modality is inexpensive, easily configurable, and straightforward to use, which would make it a cost-effective and easy solution to set up. Unlike similar studies [31, 40], the proposed system does not need many IMU sensors as it uses one BNO055 IMU. In similar studies, participants were required to wear a particular wearable object with three IMUs on their hands; however, the wearable object could be perceived as intrusive and difficult to apply by the players, particularly novice players. Besides, one of the main issues in educational assistance systems is user preference. Even high-performance systems with lower user preference would be considered failures [76]. In this work, an IMU embedded racket was used as the data collection tool, which increased the privacy (not visual) and anonymity of players' issues, thus increasing the players' confidence. Moreover, the practical and close cooperation among all the study's parties (the players, trainers, and researchers) in the design and data collection phases of the research could overcome the challenge mentioned in the recent Rajšp and Fister's study [41].

As future work, we planned to complete the system's last component, the smart feedback component. To complete the final phase of the system, facing uncertainly data challenges is inevitable. Thus, it seems that adapting methodologies based on the ellipsoid-shaped Fuzzy Inference Systems with neuro-fuzzy approach could be an appropriate solution to overcome this challenge [16]. After that, the virtual-coach Table Tennis shadow-play training system could be integrated with Cloud Internet Services to store the player's practice history for the next phase of recommendations and feedback. Besides, it can be integrated with web-based and mobile applications to show graphical and visual feedback to players and to coaches if players train with physical coaches. Long-term storage of the automatic evaluation of strokes and feedback could produce progress rates of the players' performance. The system would assist players with training Table Tennis shadow-play individually and provide appropriate automatic feedback to increase their performance without a coach.

## Figures and Tables

**Table 1:** The experiment framework phases.

#	Phase	Descriptions
**1**	Hardware setup	Selecting an appropriate sensor
		The sensor placement, the sensor calibration
**2**	Data acquisition [20]	Participants characteristics
		Defining data acquisition protocols
		Labeling and scoring samples
**3**	Preprocessing [17]	Eliminating incomplete data
		Time-series signals segmentation
		Normalizing segmented signals
**4**	Processing	Networks setup
		Model parameters setup
		Training and testing models
		Computing performance metrics
		Comparing models' performances

**Table 2:** The collected data statistical specification^*∗*^.

Type of participant	#	Samples	# Forehand strokes classified by type			Gender	Age	Duration
			Basic	Push	Top			
Professional	8	1080	360	360	360	Mixed	20–38	3 days
Novice	8	648	227	191	230	Mixed	19–22	5 days
Total	16	1728	587	551	590	—	—	8 days

^*∗*^The coaches as supervisors controlled all collected data in the limited conditions.

**Table 3:** Schema of the dataset.

Attribute	Description	Data type
Sensory data	The racket movement and orientation measurements	Numeric
Quality scores	The average scores of each criterion	Numeric
Labels	The label of the performed stroke (B, T, and P)	Alphabet

**Table 4:** Basic information of the dataset.

Dataset	Strokes' name	Samples' number	Percentage	Feature size
(1) Sensory data	Basic	740	48%	12 × 70=840
	Topspin	393	26%	
	Push	392	26%	
	Total	1525	100%	
(2) Quality scores	Criteria's name	Criteria's number	—	Range of scores value
	*C*_1_	1525	—	(0–100)%
	*C*_2_			
	*C*_3_			
	*C*_4_			
	*C*_5_			
(3) Labels	Labels' name	Labels' number	—	Percentage
	B	740	—	48%
	T	393		26%
	P	392		26%

**Table 5:** RMSE, MAPE, MAE, and ${\bar{R}}^{2}$ for the Table Tennis Forehand stroke self-collected dataset.

Strokes	Model	RMSE		MAPE	MAE	${\bar{R}}^{2}$
		Training	Test			
Basic	RBF-SVR	4.91	4.81	0.10	8.70	0.962
	**LSTM**	**4.79**	**4.44**	**0.07**	**3.30**	**0.998**
	2DCNN	8.52	6.55	0.10	20.92	0.987
Topspin	RBF-SVR	2.45	2.51	0.05	5.12	0.965
	**LSTM**	**4.33**	**2.62**	**0.04**	**2.02**	**0.999**
	2DCNN	9.35	5.04	0.11	5.30	0.987
Push	RBF-SVR	2.08	2.20	0.04	3.80	0.961
	**LSTM**	**3.75**	**2.22**	**0.02**	**2.81**	**0.996**
	2DCNN	8.84	3.84	0.09	3.67	0.900

**Table 6:** Statistical information of output data of RBF-SVR, LSTM, and 2DCNN.

Models	Strokes	Statistics	*C*_1_	*C*_2_	*C*_3_	*C*_4_	*C*_5_	Total	W. mean	W. STD
RBF-SVR	Basic	Mean	2.75	4.88	4.56	4.16	3.32	3.95	3.74	4.07
		STD	2.5	5.99	4.67	4.47	3.7	4.19		
	Top	Mean	3.94	3.96	4.77	3.98	4.36	4.12		
		STD	4.24	3.83	5.07	4.26	5.95	4.73		
	Push	Mean	2.21	2.86	3.59	3.62	2.95	3.09		
		STD	3.3	2.47	4.23	2.25	3.13	3.17		
LSTM	Basic	Mean	1.85	4.08	3.63	3.93	3.28	3.35	**3.16**	**3.6**
		STD	1.69	5.09	3.87	3.54	3.47	3.78		
	Top	Mean	3.61	3.15	4.00	3.18	3.43	3.47		
		STD	3.99	3.50	4.26	3.36	5.15	4.12		
	Push	Mean	1.78	2.61	3.26	2.81	2.05	2.50		
		STD	2.75	2.04	3.98	1.92	2.32	2.76		
2DCNN	Basic	Mean	14.61	14.43	15.25	13.13	12.17	13.92	12.10	5.97
		STD	4.72	8.95	6.77	6.24	6.28	6.82		
	Top	Mean	13.89	15.23	16.02	13.34	13.70	14.44		
		STD	7.67	7.57	6.96	6.19	5.83	6.96		
	Push	Mean	6.52	6.35	6.88	6.17	6.10	6.40		
		STD	3.59	2.95	4.08	3.43	2.85	3.42		

**Table 7:** The weighted average of RMSE, MAPE, MAE, and ${\bar{R}}^{2}$ for the Table Tennis Forehand stroke self-collected dataset.

Models	RMSE	MAPE	MAE	${\bar{R}}^{2}$
SVR	3.54	0.07	6.5	0.962
**LSTM**	**3.39**	**0.05**	**2.86**	**0.997**
2DCNN	5.12	0.10	12.37	0.963

## Data Availability

The dataset generated and analyzed during the current study is publicly available, and the data and their gathering procedures have been published earlier. Previously reported data were used to support this study, and these prior studies (and datasets) are cited at relevant places within the text as references [19–21].

## Supplementary Materials

Supplementary Materials Supplementary materials associated with this article can be found in the online version at DOI: 10.17632/b7bc9y232m.3 and DOI: 10.17632/jtv3f9pbdb.1.
