# Supplementary material for: A Deep Learning Approach for Table Tennis Forehand Stroke Evaluation System Using an IMU Sensor
Source: Comput Intell Neurosci. 2021 Apr 9;2021:5584756. doi: 10.1155/2021/5584756 (PMC8033526; doi:10.1155/2021/5584756)
Supplement: Supplementary Materials — associated with this article can be found in the online version at DOI: 10.17632/b7bc9y232m.3 and DOI: 10.17632/jtv3f9pbdb.1. [file 5584756.f1.docx]

Supplementary material associated with this article be found in the online version at https://data.mendeley.com/datasets/b7bc9y232m/3 and https://data.mendeley.com/datasets/jtv3f9pbdb/1
